# Accuracy and Short-Term Consistency of Generative AI Chatbots in Guideline-Based Endodontic Decision Support: A Multi-Model Benchmarking Study

**DOI:** 10.3390/dj14090598

**Published:** 2026-09-16

**Authors:** Ilgın İlgenli, Ezgi Avcı, Timur Köse

**Affiliations:** 1 Department of Endodontics, Faculty of Dentistry, Ege University, 35040 Izmir, Turkey; 2 Department of Biostatistics and Medical Informatics, Faculty of Medicine, Ege University, 35040 Izmir, Turkey; 3 Department of Basic Medical Sciences, Faculty of Medicine, Ege University, 35040 Izmir, Turkey

**Keywords:** large language models, chatbots, generative artificial intelligence, endodontics, root canal therapy, decision support systems, clinical, practice guidelines as topic

## Abstract

**Background/Objectives**: Large language model (LLM) chatbots are increasingly used for dental information and decision support, yet their accuracy and short-term reproducibility in endodontics remain insufficiently established. This study compared five chatbots using open-ended questions derived from established AAE and ESE endodontic guidelines. **Methods**: Twenty-six guideline-based questions were content-validated by five endodontists using Lawshe’s Content Validity Ratio. ChatGPT-4o, Gemini 2.5 Pro, DeepSeek-V3-0324, ScholarGPT (academic version built on OpenAI’s GPT-4 architecture), and MedGebra GPT-4 answered each question across three days and three sessions per day, yielding 1170 responses. Two blinded endodontists scored responses on a 5-point guideline-concordance scale. Brunner–Langer LD-F2 analyses assessed model and temporal effects, while weighted kappa evaluated response consistency. **Results**: The overall model effect was significant (*p* < 0.001). ChatGPT-4o, Gemini 2.5 Pro, DeepSeek-V3-0324, and ScholarGPT showed comparable performance, whereas MedGebra GPT-4 performed significantly lower after Bonferroni correction. The day effect was not significant (*p* = 0.054), while the session effect (*p* = 0.048) and model × session interaction (*p* = 0.030) were significant. Weighted kappa values varied across models and assessment days, ranging from 0.689–0.730 for Gemini 2.5 Pro, 0.606–0.662 for ChatGPT-4o, 0.520–0.645 for DeepSeek-V3-0324, 0.458–0.592 for ScholarGPT, and 0.240–0.739 for MedGebra GPT-4. **Conclusions**: Guideline-aligned performance and short-term reproducibility differed across the evaluated chatbots, showing that accuracy and consistency represent distinct aspects of performance. Repeated assessment captured variation missed by single-session testing. These findings support guideline-based evaluation and clinician verification when generative AI chatbots are used to provide endodontic information relevant to decision support or dental education.

## 1. Introduction

Artificial intelligence (AI) systems, particularly large language models (LLMs), are increasingly used in health sciences to support information retrieval, summarization, and decision support [1,2,3]. Across dentistry, AI applications have been explored in cancer screening, image-based diagnosis, treatment planning, and educational use cases [4,5,6]. Within endodontics, machine-learning and deep-learning methods have been investigated for periapical lesion detection, root canal morphology assessment, working length determination, and fracture identification [7,8,9,10]. In parallel, conversational LLMs (chatbots) have emerged as accessible tools for students and clinicians, with early studies evaluating their potential for knowledge transfer and formative assessment [11,12,13].

Despite their rapid adoption, comparative evidence on LLM performance in endodontics remains limited. Many studies have evaluated a single model or relied on a single testing occasion, limiting the ability to assess whether performance is reproducible over time [14,15]. Fluent and seemingly authoritative responses may still contain inaccuracies or vary when the same question is repeated, raising concerns about their reliability in clinical contexts [16]. For dental applications, therefore, evaluating whether an AI system provides a correct answer at a single time point is not sufficient; its responses should also remain reasonably consistent when the same clinical question is presented repeatedly. Meaningful evaluation also requires comparison with evidence-based clinical standards. The American Association of Endodontists (AAE) Guide to Clinical Endodontics (2019) [17] and the European Society of Endodontology (ESE) S3 Clinical Practice Guideline (2023) [18] provide complementary North American and European guidance on diagnosis, treatment, and outcome assessment in endodontics [17,18]. Together, these sources provide clinically relevant reference standards against which guideline alignment in chatbot responses can be assessed.

Beyond information retrieval, LLMs are being explored for diagnostic reasoning, differential diagnosis generation, triage assistance, and case difficulty assessment, all of which relate to clinical pathways in endodontics. Their ability to process symptoms, radiographic descriptions, and guideline-based criteria has prompted interest in their use for early-stage diagnostic support and treatment planning [11,19]. Whether their responses remain aligned with endodontic guidance across repeated use, however, requires systematic evaluation.

In dental education, LLMs have also been explored for structured learning, self-assessment, simulated clinical scenarios, and feedback generation, complementing traditional teaching methods. As digital literacy becomes increasingly relevant to dental education, understanding both the strengths and limitations of LLM-generated information is important for undergraduate education and continuing professional development [7,20,21].

Concerns remain regarding hallucination, deviation from guideline recommendations, and variation in responses over time, all of which may result in plausible but inaccurate or inconsistent information [22]. These concerns make repeated evaluation particularly relevant: a single administration measures performance at one point in time but does not establish whether the same model will provide a comparable response when the same clinical question is asked again. Accordingly, assessments that compare multiple models across repeated testing sessions provide information that single-instance evaluations cannot capture.

Recent studies illustrate both the growing interest in this topic and the methodological differences in how endodontic chatbot performance has been evaluated. Suárez et al. [11] assessed a single model at a single time point, providing an initial benchmark but not an assessment of temporal consistency or comparative reliability. Abdulrab et al. [23] subsequently compared four chatbots, including MedGebra GPT-4, across two testing rounds separated by one week using 100 multiple-choice questions derived from two endodontic textbooks. This design incorporated both temporal and multi-model comparisons, although the questions were closed-format and were not content-validated or mapped to a clinical practice guideline. Özbay et al. [24] evaluated three chatbots (Google Bard, ChatGPT-3.5, and ChatGPT-4) using 40 open-ended endodontic questions derived from ESE and AAE position statements, but each question was administered once per model, leaving temporal consistency unexamined. Büyüközer Özkan et al. [25] compared four chatbots using 20 regenerative-endodontics questions derived from ESE and AAE guidance across different languages and prompting conditions. Their study focused specifically on regenerative procedures rather than repeated administration of identical questions across separate days. de Araújo et al. [26] evaluated eleven LLMs over five repeated rounds using sixty multiple-choice questions derived from AAE and ESE position statements, demonstrating the feasibility of repeated multi-model testing, although the closed-response format differed from the open-ended format encountered when users seek explanations or recommendations from conversational AI systems.

Taken together, these studies leave a specific methodological gap. Previous research has examined individual elements of chatbot performance in endodontics, including guideline-derived questions, open-ended responses, multi-model comparisons, and repeated testing. However, based on the studies identified above, these elements have not been combined within a single design using open-ended guideline-based endodontic questions derived jointly from the AAE Guide to Clinical Endodontics (2019) [17] and the ESE S3 Clinical Practice Guideline (2023) [18], formal expert-based content validation, and repeated assessment of both response accuracy and short-term temporal consistency across multiple chatbot systems. This distinction is clinically relevant because accuracy and reproducibility address different aspects of performance: a model may provide a guideline-concordant response on one occasion without reproducing comparable performance when the same clinical question is presented again.

To address this gap, the present study used a content-validated benchmarking framework based on endodontic topics and recommendations represented in the AAE and ESE guidelines. First, the question set was developed from clinical topics and recommendations contained in the AAE Guide to Clinical Endodontics and the ESE S3 Clinical Practice Guideline and required open-ended, guideline-concordant responses rather than selection from predetermined answer choices [17,18]. Second, the questions underwent formal content validation using Lawshe’s Content Validity Ratio, with independent assessment by five endodontic specialists. Third, each chatbot was evaluated repeatedly across three separate days and three sessions per day, allowing short-term temporal consistency to be examined alongside response accuracy.

Five user-facing AI chatbot systems were evaluated: ChatGPT powered by GPT-4o, Gemini 2.5 Pro, DeepSeek Chat powered by DeepSeek-V3-0324, ScholarGPT (academic version built on OpenAI’s GPT-4 architecture), and MedGebra GPT-4 (clinically oriented decision-support model trained on Saudi Arabian medical guidelines). The primary aim was to compare the guideline-based accuracy of these systems in answering open-ended endodontic knowledge questions and to determine whether their responses remained consistent across repeated administrations. Accordingly, the following null hypotheses were tested:

**H0_1_ (Model effect).**  *There is no statistically significant difference in accuracy scores among the evaluated LLM-based chatbots.*

**H0_2_ (Short-term response consistency).**  *There is no significant variation in response consistency across repeated administrations for each evaluated LLM-based chatbot.*

## 2. Materials and Methods

### 2.1. Study Design and Reference Standards

This comparative study evaluated the performance of five user-facing generative AI chatbot systems in answering guideline-based, open-ended endodontic knowledge questions. The AAE Guide to Clinical Endodontics [17] and the ESE S3 Clinical Practice Guideline [18] served as the reference standards for question development and response assessment. Reporting followed the CHART (Chatbot Assessment Reporting Tool) statement for studies evaluating chatbot-generated health advice [27]. No human or animal participants or patient data were involved; therefore, institutional ethics approval and informed consent were not required.

### 2.2. Chatbot Systems Evaluated

Five user-facing generative AI chatbot systems were evaluated:

▪ ChatGPT powered by GPT-4o (OpenAI, San Francisco, CA, USA);
▪ Gemini 2.5 Pro (Google, Mountain View, CA, USA);
▪ DeepSeek Chat powered by DeepSeek-V3-0324 (DeepSeek AI, Hangzhou, China);
▪ ScholarGPT (academic version built on OpenAI’s GPT-4 architecture; OpenAI, San Francisco, CA, USA);
▪ MedGebra GPT-4 (clinically oriented decision-support model trained on Saudi Arabian medical guidelines, developed by Medgebra Chatbots for Medical Professionals, accessed via https://medical-chatbot.medgebra.com/, accessed on 13 September 2026).

Testing was performed on 9, 16, and 23 July 2025 in Turkey. The systems were evaluated as available through their user-facing interfaces on the testing dates. Where an interface did not display a specific build identifier, no additional build information was assigned.

### 2.3. Question Development

The question set was developed from the AAE Guide to Clinical Endodontics (2019) and the ESE S3 Clinical Practice Guideline (2023) [17,18]. Relevant sections and recommendations were reviewed to identify clinically relevant topics and decision points across nine domains. The final set included 4 questions on diagnosis, 5 on vital therapies, 3 on non-surgical root canal treatment, 2 on pulpal and periapical diseases, 3 on complications, 4 on trauma, 1 on devital bleaching, 2 on restoration, and 2 on sterilization. The questions were selected to provide coverage of clinically relevant topics and decision points represented in the source guidelines rather than an equal number of items within each domain. Accordingly, the question set was designed as a broad guideline-based benchmark and not to provide domain-specific estimates of chatbot performance. The questions were guideline-based, open-ended knowledge questions rather than patient-specific case scenarios. The study therefore assessed the ability of the chatbots to provide guideline-aligned information relevant to endodontic decision support, rather than their ability to make clinical decisions in individual patient cases. Open-ended questions were formulated to assess guideline-aligned responses regarding indications, treatment objectives and procedures, complications, and patient or case selection. Open-ended rather than fixed-choice questions were used to evaluate the content of the responses generated by each chatbot. The complete set of 26 questions is provided in Supplementary Materials (Table S1).

### 2.4. Content Validity Assessment

Content validity was assessed using Lawshe’s Content Validity Ratio (CVR) [28]. Five endodontists, each with more than five years of clinical and academic experience, independently classified each question as ‘essential,’ ‘useful but not essential,2 or ‘not necessary.’ The CVR for each item was calculated as follows:

CVR = (n_e_ − N/2)/(N/2)

where n_e_ represents the number of experts who rated the item as ‘essential’ and N represents the total number of experts.

For five experts, the minimum acceptable CVR was 1.00, corresponding to unanimous classification of an item as essential by all five experts [28,29]. All 26 questions achieved a CVR of 1.00 and were therefore retained without revision or removal. The five endodontists involved in content validation did not participate in the subsequent scoring of chatbot responses.

### 2.5. Guideline Concordance Mapping

Before chatbot testing, each question was mapped to the corresponding section or recommendation of the AAE (2019) or ESE S3 (2023) guidance [17,18]. For each item, the relevant guideline source and the key elements expected in a guideline-concordant response were defined in advance. These predefined elements provided a common reference for evaluating chatbot responses and reduced reliance on post hoc judgments during scoring. Supplementary Materials (Table S1) present the complete question set, guideline sources, corresponding sections or recommendations, predefined response elements, and CVR scores.

### 2.6. Administration Protocol

Each chatbot received the complete set of 26 questions. Testing took place on three separate days at one-week intervals, 9, 16, and 23 July 2025, with three independent sessions performed on each testing day. Thus, every question was administered nine times to each chatbot.

A new chat was opened for each session to minimize contextual carry-over between sessions. Chat histories and local caches were cleared before testing. Questions were presented verbatim and in the same order to all five systems, and all responses were obtained in English. No follow-up prompts or conversational clarification were used. The complete prompt set is provided in Supplementary Materials (Table S1).

The protocol generated 1170 responses in total: 26 questions × 5 chatbots × 3 testing days × 3 sessions. (26 questions × 5 chatbots × 3 days × 3 sessions).

### 2.7. Response Scoring and Rater Procedure

Two experienced endodontists independently evaluated each response using a five-point accuracy scale, with 5 representing a completely correct and comprehensive response and 1 representing an incorrect or irrelevant response. Given the broad and open-ended nature of the questions, chatbot responses could cover multiple aspects of a topic. To reduce the possibility that response breadth alone would be interpreted as guideline concordance, ratings were based on the ‘Predefined Key Elements for Correct Response,’ which were derived from the same AAE and ESE guidance documents used to develop the questions and are provided in Table S1. Ratings also considered contextual relevance, accuracy, and completeness. The anchoring criteria were informed by a modified Global Quality Score (GQS) [30] framework, which was used to structure the qualitative dimensions of assessment rather than as a separate numerical outcome.

To reduce the potential influence of model recognition on scoring, chatbot outputs were anonymized and randomized before evaluation, and the raters were blinded to model identity. Initial disagreements were resolved by consensus using the ‘Predefined Key Elements for Correct Response’ as the reference standard. Only the final consensus score was retained for analysis. Consequently, inter-rater reliability statistics for the two independent initial ratings were not calculated.

**Figure 1:**
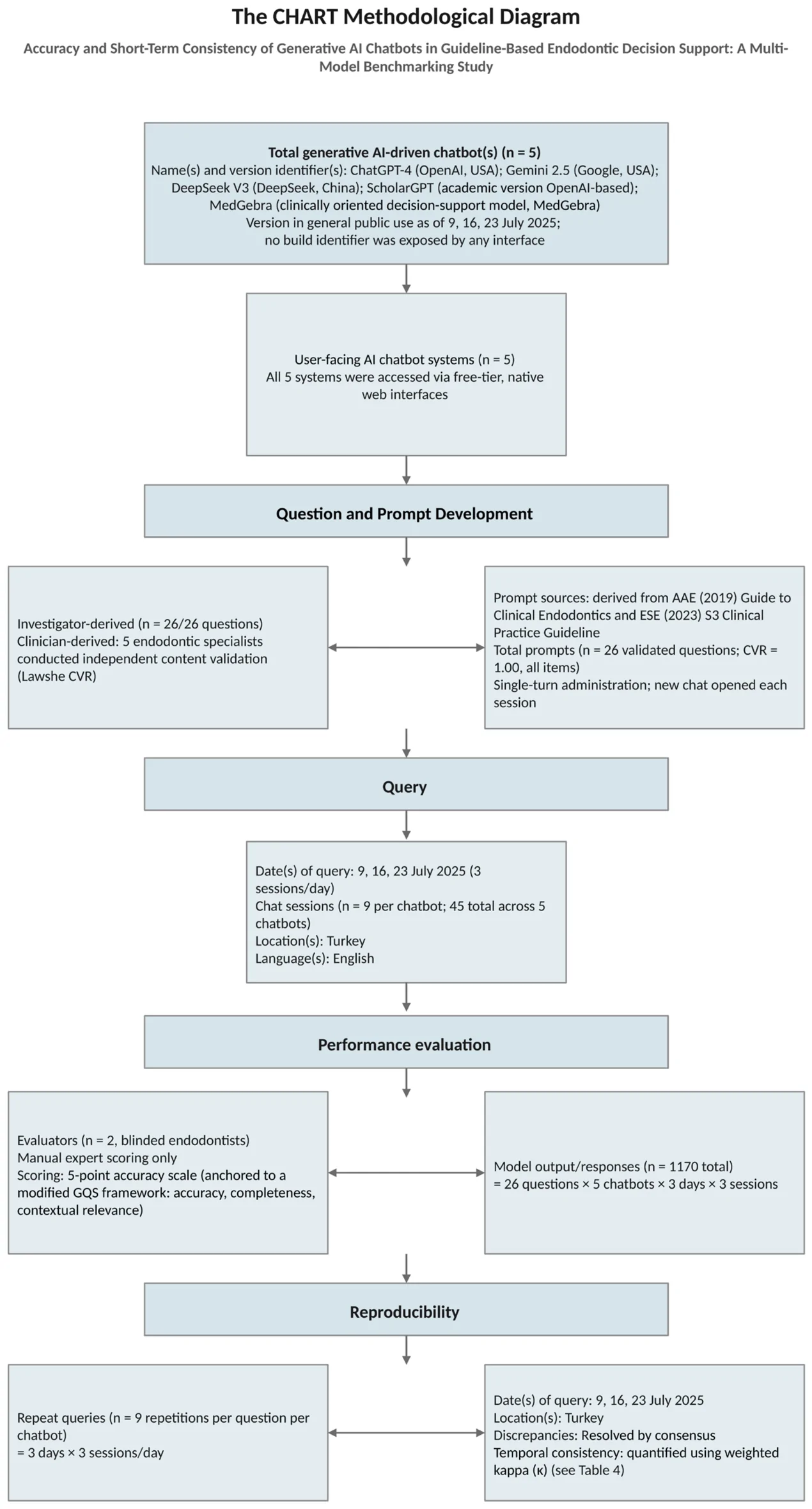
Study workflow mapped to the CHART (Chatbot Assessment Reporting Tool) methodological diagram [17,18,27].

A schematic representation of the study design and assessment workflow is provided in Figure 1.

### 2.8. Statistical Analysis

Because the outcome consisted of repeated ordinal accuracy scores and did not require an assumption of normality, rank-based nonparametric methods were used. Between-model differences and temporal effects were evaluated using the Brunner–Langer LD-F2 model in R version 4.3.1 (R Foundation for Statistical Computing, Vienna, Austria). The individual question was treated as the unit of analysis (*n* = 26), with repeated observations obtained across chatbot systems and testing occasions.

The primary LD-F2 analyses examined the effects of chatbot model, time, and the model × time interaction. ANOVA-type statistics from the LD-F2 models were used for inference. Pairwise chatbot analyses were subsequently performed where applicable. For each pairwise comparison, Bonferroni adjustment was applied across the three effects examined within that comparison, namely model, time, and model × time interaction; adjusted *p*-values were calculated as the raw *p*-value multiplied by three and capped at 1.00.

Short-term response consistency across repeated assessments was examined separately using weighted kappa coefficients. Weighted κ values were interpreted using the Landis–Koch benchmarks [31]. Statistical significance was set at *p* < 0.05.

## 3. Results

### 3.1. Overall Model Effects

**Figure 2:**
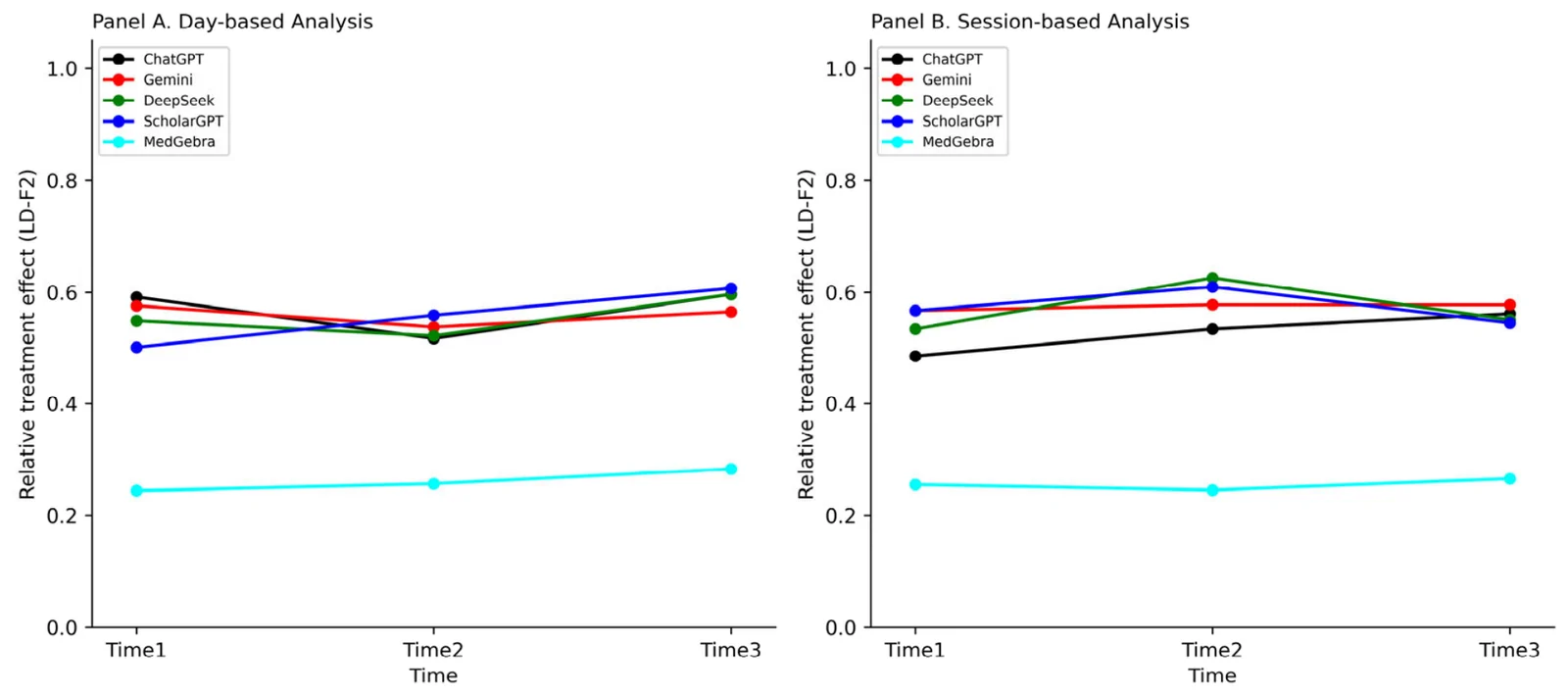
Relative treatment effects of five generative AI chatbots for guideline-based endodontic decision support. (**A**) Day-based analysis across three independent testing days. (**B**) Session-based analysis across three repeated sessions. The *y*-axis shows the relative treatment effect (RTE) estimated using the nonparametric LD-F2 model, and the *x*-axis represents the three assessment days or sessions. ChatGPT-4o, Gemini 2.5 Pro, DeepSeek-V3-0324, and ScholarGPT showed broadly overlapping relative treatment effects, whereas MedGebra showed consistently lower values in both analyses.

The comparative performance of the five user-facing AI chatbot systems on the guideline-based endodontic question set is summarized in Table 1 and Figure 2.

The omnibus Brunner–Langer LD-F2 analysis identified a statistically significant model effect in both the day-based and session-based analyses (both *p* < 0.001), showing that guideline-based response performance differed among the five chatbot systems. Pairwise comparisons showed no statistically significant model effects among ChatGPT-4o, Gemini 2.5 Pro, DeepSeek-V3-0324, and ScholarGPT after Bonferroni correction. In contrast, each comparison involving MedGebra showed a significant model effect, with MedGebra having lower relative treatment effects than the other four systems (Table 2).

**Figure 3:**
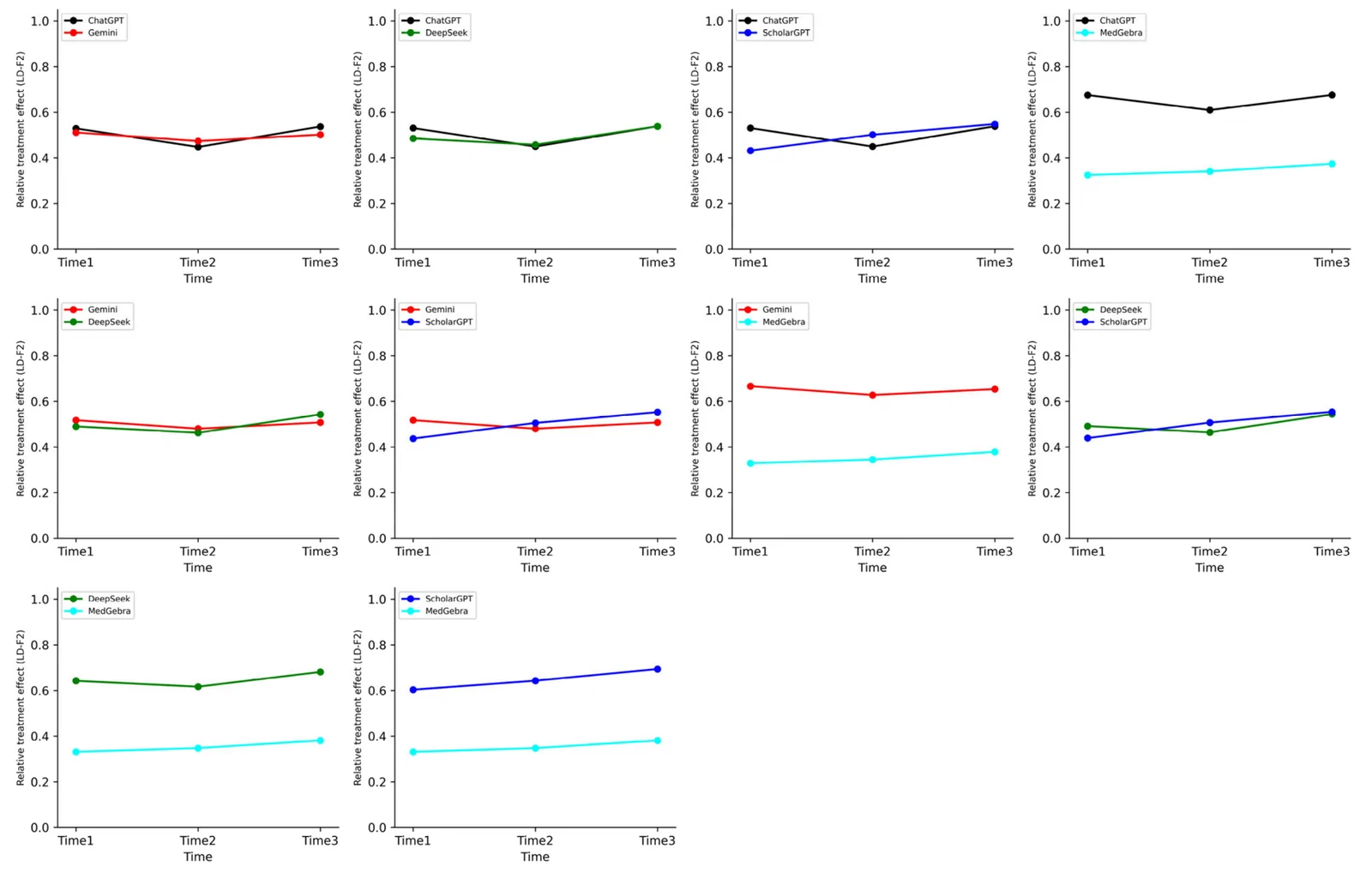
Pairwise comparisons of generative AI chatbot performance across the three assessment days. Relative treatment-effect trajectories are shown for all pairwise comparisons among ChatGPT-4o, Gemini 2.5 Pro, DeepSeek-V3-0324, ScholarGPT, and MedGebra. The *y*-axis represents the relative treatment effect estimated using the LD-F2 model, and the *x*-axis represents the three assessment days (Time 1–3). Comparisons among ChatGPT-4o, Gemini 2.5 Pro, DeepSeek-V3-0324, and ScholarGPT showed largely overlapping trajectories, whereas comparisons involving MedGebra showed consistently lower relative treatment effects for MedGebra.

Pairwise post-hoc analyses therefore localized the overall between-model difference to comparisons involving MedGebra. No significant model effects were found among ChatGPT-4o, Gemini 2.5 Pro, DeepSeek-V3-0324, and ScholarGPT after correction for multiple comparisons (Table 2 and Table 3; Figure 3). This pattern shows that the principal between-model difference in guideline-based endodontic response performance arose from the lower relative treatment effects observed for MedGebra.

### 3.2. Temporal Effects

No statistically significant overall change was observed across the three assessment days (*p* = 0.054), and the model × day interaction was also non-significant (*p* = 0.295). Thus, the data did not provide evidence of an overall day effect or of differences among models in their day-to-day trajectories during the study period.

In the session-based analysis, however, both the session effect (*p* = 0.048) and model × session interaction (*p* = 0.030) were statistically significant. These findings indicate that response performance varied across repeated within-day sessions and that the pattern of this variation differed among chatbot systems.

In the pairwise day-based analyses, the ChatGPT-4o–DeepSeek-V3-0324 comparison produced a raw *p*-value of 0.034 for the day effect. After Bonferroni correction across the three effects tested within this comparison, the corrected *p*-value was 0.101 and was therefore not statistically significant (Table 3). No other corrected pairwise day effects reached statistical significance.

### 3.3. Short-Term Response Consistency

Weighted kappa coefficients were calculated separately for each chatbot across the three assessment-day pairs to describe within-model short-term response consistency (Table 4). Gemini 2.5 Pro showed pairwise coefficients ranging from κ = 0.689 to 0.730, while the corresponding ranges were κ = 0.606–0.662 for ChatGPT-4o, κ = 0.520–0.645 for DeepSeek-V3-0324, and κ = 0.458–0.592 for ScholarGPT. MedGebra showed coefficients ranging from κ = 0.240 to 0.739.

**Figure 4:**
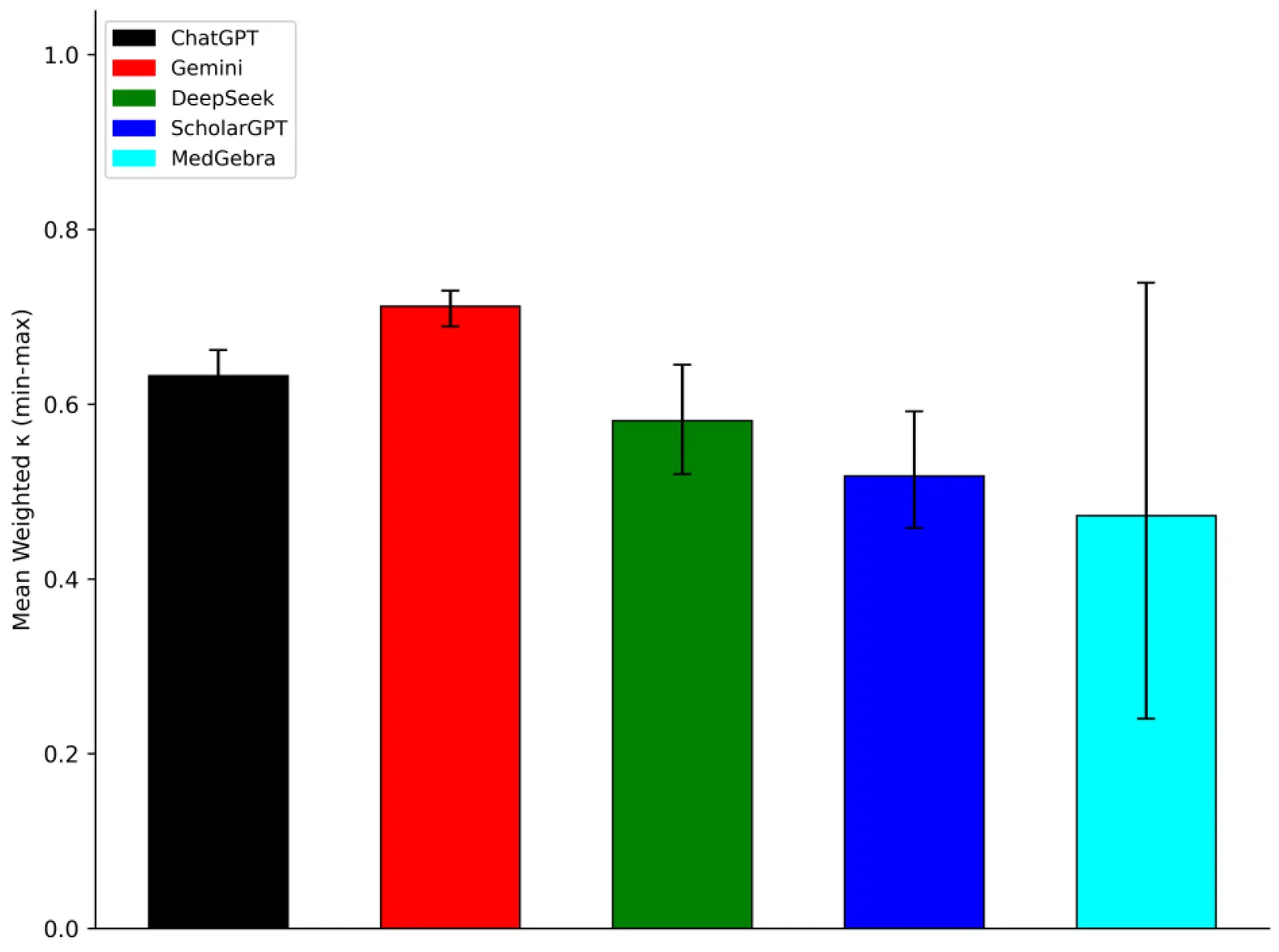
Short-term response consistency of the five generative AI chatbot systems across assessment days. Bars represent the mean pairwise weighted kappa (κ) coefficient for each chatbot, and whiskers show the observed minimum–maximum range across the three day-pair comparisons (G1–G2, G1–G3, and G2–G3).

All pairwise weighted kappa coefficients were statistically significant at *p* ≤ 0.001 except the MedGebra G2–G3 comparison (κ = 0.240, 95% CI: −0.107 to 0.587; *p* = 0.054). These coefficients describe within-model agreement across assessment-day pairs and should be interpreted separately from the between-model performance differences identified by the LD-F2 analysis. The mean weighted kappa coefficients and their observed minimum–maximum ranges are presented in Figure 4.

## 4. Discussion

Previous studies have evaluated chatbot responses using questions derived from AAE and ESE position statements; however, we identified no previous study that combined the AAE Guide to Clinical Endodontics [17] and the ESE S3 Clinical Practice Guideline [18] as reference frameworks across multiple endodontic decision domains while also using open-ended, content-validated questions and repeated testing across both days and sessions. Related studies have incorporated individual elements of this design. Abdulrab et al. [23] evaluated several chatbots, including MedGebra, at two time points using closed-format textbook-based rather than guideline-based questions, without assessing within-day variation. Özbay et al. [24] used an open-ended, guideline-derived question set validated with Lawshe’s CVR, but administered each question only once per model. Büyüközer Özkan et al. [25] evaluated open-ended, guideline-derived questions across languages and prompt conditions, focusing specifically on regenerative endodontics rather than repeated testing across separate days. de Araújo et al. [26] conducted five repeated rounds with eleven models using AAE- and ESE-derived questions but used a multiple-choice format and did not report formal content validation using a method such as Lawshe’s CVR. The present study therefore extends previous endodontic chatbot research by integrating guideline mapping, formal content validation, open-ended questions, five chatbot systems, and repeated multi-day and within-day assessments within a single design, allowing guideline-aligned performance and short-term response reproducibility to be evaluated as distinct but related aspects of chatbot behavior.

The findings directly address the study hypotheses. The null hypothesis for the model effect was rejected. The omnibus Brunner–Langer LD-F2 analysis identified a significant model effect (*p* < 0.001). Pairwise analyses showed significantly lower relative treatment effects for MedGebra than for ChatGPT-4o, Gemini 2.5 Pro, DeepSeek-V3-0324, and ScholarGPT after Bonferroni correction, whereas no significant pairwise model effects were detected among the other four systems. These results indicate that the overall model effect was largely associated with the lower relative performance observed for MedGebra rather than broad differences among all five systems. This finding should be interpreted considering differences in the scope and intended use of the evaluated systems. MedGebra is described as a clinically oriented system trained on Saudi Arabian medical guidelines, whereas responses in the present study were evaluated specifically against recommendations from the AAE Guide to Clinical Endodontics and the ESE S3 Clinical Practice Guideline. This difference in reference context may have contributed to MedGebra’s lower concordance with the predefined AAE/ESE-derived criteria. Its lower performance in this benchmark should therefore be interpreted as lower concordance with the specific endodontic reference standards used in this study rather than as evidence of poorer clinical performance in other settings or against the guidance for which the system was developed.

The hypothesis concerning short-term response consistency was also not supported uniformly across the evaluated systems. Weighted κ values differed across models and assessment-day comparisons. Gemini 2.5 Pro showed consistently high pairwise agreement within the observed data (κ = 0.689–0.730), while ChatGPT-4o (κ = 0.606–0.662), DeepSeek-V3-0324 (κ = 0.520–0.645), and ScholarGPT (κ = 0.458–0.592) showed different levels of agreement across assessment days. MedGebra showed the widest range (κ = 0.240–0.739), including a non-significant G2–G3 agreement (κ = 0.240, 95% CI: −0.107 to 0.587; *p* = 0.054). These findings indicate that repeated responses were not equally reproducible across models or assessment-day comparisons. Importantly, the weighted κ results describe within-model agreement over time and should not be interpreted as a formal statistical comparison of reproducibility between models.

The repeated-measures analysis further distinguished between day-level and within-day temporal effects. No statistically significant overall day effect was detected (*p* = 0.054), and the model × day interaction was also non-significant (*p* = 0.295). In contrast, the session effect (*p* = 0.048) and model × session interaction (*p* = 0.030) were statistically significant. The session effect was close to the conventional significance threshold and should therefore be interpreted cautiously. Taken together, these findings provide evidence of some within-day variation and indicate that the pattern of session-related variation was not uniform across chatbot systems, while providing no statistically significant evidence of an overall change across the three assessment days. The magnitude and clinical relevance of these short-term differences remain uncertain. Repeated administration was an intentional feature of the study design because variation in responses to identical questions was itself a feature being evaluated. At the same time, the observed consistency estimates reflect only the number and timing of repetitions included in this protocol and may not capture the full range of response variability that could occur with additional administrations or over longer periods. Complementing these temporal analyses, the weighted kappa coefficients characterized within-model agreement across the three assessment-day pairs. Gemini showed κ values of 0.689–0.730 across the three comparisons, while the corresponding ranges were 0.606–0.662 for ChatGPT, 0.520–0.645 for DeepSeek, and 0.458–0.592 for ScholarGPT. MedGebra showed the widest observed range (κ = 0.240–0.739), including one comparison that did not reach statistical significance.

For dental education, this distinction is relevant because a chatbot that provides an appropriate guideline-aligned response on one occasion does not necessarily provide an equivalent response when the same question is presented again. As AI-assisted learning becomes increasingly relevant to dental education, students and clinicians need to evaluate generated information critically and verify clinically consequential content against appropriate evidence-based sources. The present findings support supervised rather than uncritical use of generative AI for guideline-based endodontic learning and information retrieval [32].

These findings are also relevant when considering the potential use of LLM-based chatbots for endodontic decision support. LLM-based chatbots have been investigated for patient communication, diagnostic reasoning, and related clinical applications [11,12,13]. The present study, however, evaluated guideline concordance and short-term response consistency rather than diagnostic or treatment-planning performance in actual patient cases. A model may produce responses that score well against guideline-derived criteria while still showing variation when the same questions are repeated. This distinction is important when considering clinically consequential uses of generative AI. The present findings do not provide a basis for autonomous diagnostic or treatment-planning use of the evaluated chatbots, and outputs that may influence clinical decisions should be verified by a qualified clinician against current evidence and clinical guidelines [22].

Several methodological features strengthen the interpretation of these findings. The use of open-ended questions derived from two established endodontic guidance documents, formal content validation using Lawshe’s CVR, blinded assessment against predefined guideline-derived criteria, and repeated administration across three days and three sessions per day provided a structured framework for evaluating both guideline-aligned performance and short-term response consistency. The Brunner–Langer LD-F2 framework was used for the repeated-measures analysis, and the study workflow was reported according to the CHART framework [27].

Recent studies have also raised concerns regarding variability and inaccurate or fabricated information in generative AI outputs [33,34]. Findings across dental studies have not been uniform, in part because evaluations differ in chatbot versions, question formats, reference standards, prompting procedures, and assessment methods [11,34,35,36,37]. The present results further support treating guideline-aligned performance and short-term within-model response reproducibility as distinct aspects of chatbot evaluation. This distinction is particularly relevant when generative AI is evaluated for endodontic diagnosis, case assessment, or treatment-related decision support [38]. Within the present dataset, MedGebra showed lower relative treatment effects than the other evaluated systems, while its within-model weighted κ estimates ranged from 0.240 to 0.739. Previous dental studies have also included MedGebra in comparative assessments [39,40]; however, differences in study design, question content, reference standards, and evaluation criteria should be considered when comparing results across studies.

This study has several limitations. Because the 2019 AAE Guide to Clinical Endodontics was included in the reference framework, the findings were evaluated in the context of the recommendations provided in this guideline. The 26 questions were not evenly distributed across the nine endodontic domains; therefore, the findings are best interpreted as reflecting performance across the overall guideline-based question set. The questions were also designed to assess guideline-based knowledge rather than patient-specific clinical scenarios requiring the integration of multiple diagnostic and prognostic variables. Although both evaluators initially scored all responses independently, only the final consensus scores were retained. Consequently, inter-rater reliability could not be quantified, limiting the assessment of scoring reliability for the open-ended responses. The study was conducted exclusively in English and on three predefined assessment dates, which limits the generalizability of the findings to other languages and longer time periods. Although repeated administration was intentionally used to characterize short-term response consistency, the resulting estimates reflect repetitions conducted within a defined assessment period. Longer observation periods may reveal additional variability and yield different estimates of short-term consistency. Another consideration is the dynamic nature of chatbot systems, which are continuously updated. Changes to the underlying models or user interfaces after the study period may affect their performance. In addition, specific model build identifiers were not publicly available through some interfaces, making exact replication of the model versions tested in this study more difficult.

## 5. Conclusions

This study identified between-model differences in guideline-aligned performance and characterized short-term within-model response reproducibility across repeated assessments. ChatGPT-4o, Gemini 2.5 Pro, DeepSeek-V3-0324, and ScholarGPT performed similarly overall, whereas MedGebra showed significantly lower performance. Within-model response consistency varied across assessment periods, reinforcing the distinction between guideline-aligned performance and short-term reproducibility. The multi-model, repeated-session design identified short-term variation that could not be assessed in a single administration and allowed both performance and short-term reproducibility to be examined over time. The findings support systematic, guideline-based evaluation of generative AI chatbots and clinician verification of their outputs when these systems are used to provide endodontic information relevant to clinical decision support or dental education.

Future studies should assess these systems over longer periods and in different languages, use more targeted and focused questions to assess specific aspects of guideline concordance, and incorporate scenarios that more closely reflect clinical practice. Given the continuing evolution of generative AI systems, future research should also reassess their performance over time and examine the clinical and educational relevance of both guideline-aligned performance and short-term response reproducibility.

## Figures and Tables

**Table 1:** Omnibus Brunner–Langer LD-F2 results for model and time (day/session) effects.

Effect	Test Statistic (Day/Session)	df (Day/Session)	*p*-Value (Day/Session)	Interpretation
Model	29.613/25.931	2.72/2.87	<0.001/<0.001	Significant model effect in both analyses
Time	2.997/3.426	1.87/1.44	0.054/0.048	No day effect; significant session effect
Model × Time	1.221/2.434	5.19/5.22	0.295/0.030	No day interaction; significant session interaction

**Table 2:** Pairwise post-hoc comparisons of model, day, and model × day effects with Bonferroni-corrected *p*-values.

Comparison	Model Effect		Day Effect		Model × Day		Interpretation
	Raw *p*	Bonferroni-Corrected *p*	Raw *p*	Bonferroni-Corrected *p*	Raw *p*	Bonferroni-Corrected *p*	
ChatGPT-4o—Gemini 2.5 Pro	0.789	1.000	0.067	0.201	0.479	1.000	No significant difference
ChatGPT-4o—DeepSeek-V3-0324	0.617	1.000	0.034	0.101	0.567	1.000	No significant difference
ChatGPT-4o—ScholarGPT	0.628	1.000	0.071	0.212	0.053	0.160	No significant difference
ChatGPT-4o—MedGebra	<0.001	<0.001	0.168	0.505	0.392	1.000	ChatGPT-4o performed significantly higher
Gemini 2.5 Pro—DeepSeek-V3-0324	0.917	1.000	0.247	0.740	0.369	1.000	No significant difference
Gemini 2.5 Pro—ScholarGPT	0.929	1.000	0.230	0.689	0.050	0.149	No significant difference
Gemini 2.5 Pro—MedGebra	<0.001	<0.001	0.600	1.000	0.315	0.945	Gemini 2.5 Pro performed significantly higher
DeepSeek-V3-0324—ScholarGPT	1.000	1.000	0.065	0.196	0.212	0.636	No significant difference
DeepSeek-V3-0324—MedGebra	<0.001	<0.001	0.187	0.562	0.722	1.000	DeepSeek-V3-0324 performed significantly higher
ScholarGPT—MedGebra	<0.001	<0.001	0.078	0.235	0.743	1.000	ScholarGPT performed significantly higher

**Table 3:** Full pairwise Brunner–Langer LD-F2 test statistics with Bonferroni-corrected *p*-values.

Comparison	Effect	Test Statistic	df	*p*-Value	Bonferroni-Corrected *p*
ChatGPT-4o—Gemini 2.5 Pro	Model effect	0.072	1.00	0.789	1.000
	Day effect	2.773	1.84	0.067	0.201
	Model × Day	0.704	1.78	0.479	1.000
ChatGPT-4o—DeepSeek-V3-0324	Model effect	0.250	1.00	0.617	1.000
	Day effect	3.431	1.94	0.034	0.101
	Model × Day	0.489	1.56	0.567	1.000
ChatGPT-4o—ScholarGPT	Model effect	0.235	1.00	0.628	1.000
	Day effect	2.733	1.81	0.071	0.212
	Model × Day	2.957	1.95	0.053	0.160
ChatGPT-4o—MedGebra	Model effect	51.781	1.00	<0.001	<0.001
	Day effect	1.812	1.75	0.168	0.505
	Model × Day	0.894	1.65	0.392	1.000
Gemini 2.5 Pro—DeepSeek-V3-0324	Model effect	0.011	1.00	0.917	1.000
	Day effect	1.401	1.85	0.247	0.740
	Model × Day	0.988	1.90	0.369	1.000
Gemini 2.5 Pro—ScholarGPT	Model effect	0.0078	1.00	0.929	1.000
	Day effect	1.471	1.99	0.230	0.689
	Model × Day	3.007	1.99	0.050	0.149
Gemini 2.5 Pro—MedGebra	Model effect	43.173	1.00	<0.001	<0.001
	Day effect	0.471	1.76	0.600	1.000
	Model × Day	1.084	1.30	0.315	0.945
DeepSeek-V3-0324—ScholarGPT	Model effect	0.000	1.00	1.000	1.000
	Day effect	2.790	1.86	0.065	0.196
	Model × Day	1.564	1.74	0.212	0.636
DeepSeek-V3-0324—MedGebra	Model effect	59.258	1.00	<0.001	<0.001
	Day effect	1.693	1.78	0.187	0.562
	Model × Day	0.291	1.78	0.722	1.000
ScholarGPT—MedGebra	Model effect	51.931	1.00	<0.001	<0.001
	Day effect	2.577	1.92	0.078	0.235
	Model × Day	0.244	1.67	0.743	1.000

**Table 4:** Pairwise weighted kappa coefficients for short-term response consistency across the three assessment days for each generative AI chatbot system.

AI Model	Timing Comparison	Weighted κ	SE	Z	*p*-Value	95% CI
ChatGPT-4o	G1–G2	0.606	0.148	4.150	<0.001	0.316–0.896
	G1–G3	0.662	0.155	4.327	<0.001	0.358–0.966
	G2–G3	0.630	0.131	4.486	<0.001	0.374–0.886
Gemini 2.5 Pro	G1–G2	0.730	0.115	4.995	<0.001	0.504–0.956
	G1–G3	0.717	0.127	4.695	<0.001	0.469–0.966
	G2–G3	0.689	0.118	4.742	<0.001	0.457–0.921
DeepSeek-V3-0324	G1–G2	0.579	0.142	4.015	<0.001	0.301–0.857
	G1–G3	0.520	0.157	3.547	<0.001	0.212–0.829
	G2–G3	0.645	0.134	4.571	<0.001	0.381–0.908
ScholarGPT	G1–G2	0.458	0.146	3.443	0.001	0.171–0.745
	G1–G3	0.504	0.161	3.633	<0.001	0.188–0.821
	G2–G3	0.592	0.146	4.167	<0.001	0.306–0.879
MedGebra	G1–G2	0.739	0.098	5.873	<0.001	0.547–0.932
	G1–G3	0.438	0.149	3.566	<0.001	0.147–0.730
	G2–G3	0.240	0.177	1.924	0.054	−0.107–0.587

Note: G1, G2, and G3 represent the first, second, and third assessment days, respectively. SE, standard error; CI, confidence interval.

## Data Availability

The original contributions presented in this study are included in the article/Supplementary Material. Further inquiries can be directed to the corresponding author.

## Supplementary Materials

The following supporting information can be downloaded at https://www.mdpi.com/article/10.3390/dj14090598/s1, Table S1: Guideline-based endodontic questions, corresponding clinical domains and guideline sources, predefined key elements for correct responses, and content validity ratio (CVR) scores.

## Abbreviations

The following abbreviations are used in this manuscript:

AI	Artificial intelligence
LLM	Large language model
AAE	American Association of Endodontists
ESE	European Society of Endodontology
CVR	Content Validity Ratio
CHART	Chatbot Assessment Reporting Tool
GQS	Global Quality Score
