# Supplementary material for: Accuracy and Short-Term Consistency of Generative AI Chatbots in Guideline-Based Endodontic Decision Support: A Multi-Model Benchmarking Study
Source: Dent J (Basel). 2026 Sep 16;14(9):598. doi: 10.3390/dj14090598 (PMC13605143; doi:10.3390/dj14090598)
Supplement: Supplementary file 1 [file dentistry-14-00598-s001.zip › dentistry-4556900-supplementary.pdf]

Supplementary Materials

Table S1. Guideline-based endodontic questions, corresponding clinical domains and guideline sources, predefined key elements for correct responses, and content validity ratio (CVR) scores.

QNo	Verbatim Question	Clinical Domain	Guideline Source	Section / Recommendation	Predefined Key Elements for Correct Response	CVR (n=5)
1	What tests are used in an endodontic examination?	Diagnosis	AAE 2019	Sec A	Thermal and electrical tests, cavity test, anesthesia, percussion, palpation and mobility, periodontal examination/evaluation, transillumination, observation of occlusal discrepancies.	**1**
2	What radiographic techniques are used for endodontic diagnosis?	Diagnosis	AAE 2019	Sec A	PA radiographs, angled views, bitewings, occlusal plane films, radiographs of the contralateral and opposing teeth, CBCT indications.	**1**
3	What should treatment records specific to endodontic patients include?	Diagnosis	AAE 2019	Sec B	Chief complaint documented in patient's own words, complete medical and dental history, results of diagnostic tests and clinical examination, pulpal and periradicular diagnoses with treatment recommendations, description of treatment rendered, including pulpal status at entry, prognosis communicated to the patient, recommendations for coronal restoration, preoperative, working, postoperative and follow-up radiographic records.	**1**
4	What should be the follow-up period after vital pulp therapy or non-surgical endodontic treatments?	Diagnosis	ESE S3 2023	Expert Evidence-Based Rec No7	Follow-up after vital pulp therapy: clinical history and examination at 6 months, periapical radiograph at 1 year, follow-up after nonsurgical or surgical treatment: minimum 1 year, continued up to 4 years, indicators of favourable outcome: no pain, swelling or other symptoms, no sinus tract, no loss of function. Radiographic evidence of normal periodontal ligament space.	**1**
5	What is the pulpotomy procedure and in	Vital Therapies	AAE 2019	Sec C2	Removal of coronal pulp, indicated in reversible/early irreversible pulpitis, bleeding control. Clinical indications: mechanical	**1**

	which case is it indicated?				exposure of vital pulp in primary teeth with reversible/early irreversible pulpitis, emergency procedure in permanent teeth until full root canal treatment is possible, interim procedure for immature permanent teeth to permit continued root development (apexogenesis).	
6	What is endodontic recalcification therapy and what steps are required for its application?	Vital Therapies	AAE 2019	Sec D4 Part III	Treatment or prevention of external and internal resorptive defects perforating to external tooth surface. Treatment is initiated by cleaning and shaping the canals (and possibly obturating the canals) and packing the area with appropriate material to create resorptive repair	**1**
7	What are the clinical indications for direct pulp capping?	Vital Therapies	AAE 2019	Sec D5	Mechanical exposure of a vital and asymptomatic pulp. Controlled bleeding at the exposure site. Exposure allows direct contact between capping material and vital pulp. Exposure occurs under dental dam isolation. Ability to provide an adequate coronal seal with the final restoration.	**1**
8	What are the clinical indications for regenerative endodontic procedures?	Vital Therapies	AAE 2019	Sec D4 Part I	Incomplete root development and open apex. Alternative treatments besides extraction limited to apexification or regenerative therapy. Apexogenesis not suitable because the pulp is necrotic.	**1**
9	What are the objectives of regenerative endodontic treatment?	Vital Therapies	AAE 2019	Sec D4 Part I	Treat apical periodontitis. Radiographic evidence of apical closure without supporting tissue breakdown. Retain dentition, especially during craniofacial development. Promote root development.	**1**
10	What are the clinical indications for root canal treatment?	Non-surgical RCT	AAE 2019	Sec D3	Symptomatic or asymptomatic irreversible pulpitis, with or without periapical disease, necrotic pulp, with or without periradicular disease, teeth where pulp health will be compromised during dental procedures (e.g., deep caries removal, overdenture abutments, malposed teeth, root resection), need for post placement to retain a fixed restoration, cracked or fractured teeth with pulpal involvement that can maintain periodontal health, persistent	**1**

					thermal hypersensitivity after unsuccessful alternative management.	
11	What are the objectives of root canal treatment?	Non-surgical RCT	AAE 2019	Sec D3	Alleviate present and prevent future adverse clinical signs or symptoms. Debridement and shaping of the root canal system. Create the radiographic appearance of a well-obturated root canal system where the root canal filling extends as close as possible to the apical constriction of each canal. Maintenance or promotion of periradicular tissue healing	**1**
12	Is endodontic treatment effective for emergency management of symptomatic pulpitis or apical periodontitis?	Non-surgical RCT	ESE S3 2023	Expert Evidence-Based Rec No3	Tooth retention as standard of care for symptomatic pulpitis, symptomatic apical periodontitis, and apical abscess. Provide dental treatment, not antibiotics or extraction. Acceptable emergency interventions: vital pulp therapy, pulpectomy to relieve discomfort.	**1**
13	Can pulpitis be successfully managed and the tooth preserved?	Pulpal/Periapical Diseases	ESE S3 2023	Expert Evidence-Based Rec No1	In the long-term management of pulpitis and apical periodontitis, retention of the natural dentition with adequate treatment, whenever possible. Retention postpones the need for prosthodontic replacement (fixed or removable).	**1**
14	Can apical periodontitis be successfully managed and the tooth preserved?	Pulpal/Periapical Diseases	ESE S3 2023	Expert Evidence-Based Rec No2	Restorable teeth should be preserved, not extracted. Root canal treatment is the recommended primary therapy. Appropriate coronal restoration must follow RCT to ensure long-term success. Supportive postoperative care and clinical/radiographic follow-up are required.	**1**
15	What is the appropriate management protocol for file fracture during root canal treatment?	Complications	AAE 2019	Sec D3	Assess removability or bypass feasibility of the separated fragment. If removal/bypass risks structural damage, obturate the remaining accessible canal space with a biocompatible material. Inform the patient about the instrument separation. Recall and monitor clinically and radiographically.	**1**
16	What are the treatment goals in repairing perforations during	Complications	AAE 2019	Sec D5	Seal the root canal space from the external root surface. Minimize extrusion of the repair material during placement. Promote	**1**

	root canal therapy?				periodontal tissue healing at the perforation site.	
17	What are traumatic dental injuries?	Trauma	AAE 2019	Sec F	Enamel fracture, crown fracture without pulp exposure, crown fracture with pulp exposure, crown–root fracture, root fracture, luxation injuries, avulsion, alveolar bone fracture.	**1**
18	What is the treatment protocol for complicated crown fractures?	Trauma	AAE 2019	Sec F3	Immature teeth: vital pulp therapy options: pulp capping or shallow pulpotomy, bonded final restoration or reattachment of the fractured fragment, after full root maturation, if necrosis develops nonsurgical RCT and crown placement. Permanent teeth: if a crown is not required: vital pulp therapy followed by bonded composite or fragment reattachment, provided no signs of irreversible pulpitis and contamination is minimized, if a crown is required for function or esthetics: nonsurgical RCT indicated.	**1**
19	What is the treatment protocol for traumatic root fractures?	Trauma	AAE 2019	Sec F5	Emergency management focuses on reduction and stabilization of the fracture site. Definitive care involves periodic radiographic and clinical monitoring. If pulpal necrosis develops, root canal treatment is indicated. If the fracture is apical and necrosis occurs, the apical segment may be surgically removed, either before or after nonsurgical RCT. If coronal structure is lost apical to the crestal bone, options include orthodontic extrusion or surgical crown lengthening.	**1**
20	What is the treatment protocol for dental avulsion injuries?	Trauma	AAE 2019	Sec F7	< 1-hour extra-oral dry time (or stored in acceptable medium): Rinse tooth with sterile saline, irrigate socket, replant gently. Nonrigid splint for 10–14 days. Systemic antibiotics and 0.12% chlorhexidine rinse. Immature teeth: doxycycline soaking before replantation; possibility of revascularization. If revascularization fails: apexification or regenerative treatment. Mature teeth: perform RCT within 7–10 days, including short-term calcium hydroxide. 1-	**1**

					hour extra-oral dry time: Remove necrotic PDL, immerse root in sodium fluoride for ~20 min. Irrigate socket and replant; non-rigid splint for 4–6 weeks. Systemic antibiotics; evaluate tetanus status. Mature teeth: perform timely RCT to limit resorption. Immature teeth with >1-hour dry time: not recommended for replantation.	
21	What is the intracanal bleaching protocol, and in which case is it indicated?	Devital Bleaching	AAE 2019	Sec G	Intracoronal bleaching uses oxidizing agents placed inside the coronal portion of an endodontically treated tooth. Indicated when discoloration originates from an internal source. Requires the presence of an adequate/acceptable root canal treatment prior to bleaching (whenever feasible)	**1**
22	What precautions should be taken to prevent cervical resorption during devital bleaching?	Complications	AAE 2019	Sec G	Placement of a cervical cement barrier to prevent oxidizing agent penetration into dentinal tubules in the cervical area to reduce the potential for resorption. Avoidance of 30% hydrogen peroxide combined with heat.	**1**
23	What are the indications for placing an intracanal post?	Restoration	AAE 2019	Sec H1	Inadequate remaining coronal tooth structure for restoration retention, sufficient root length to place a post while preserving the apical seal, presence of enough tooth structure to allow creation of a ferrule after crown preparation.	**1**
24	What should be considered when placing a post?	Restoration	AAE 2019	Sec. H1	Position the post along the long axis of the root, avoid perforation and root fracture, maintain the apical seal, ensure no voids between the post and surrounding dentin/root filling, place the post in root structure adequately supported by bone, minimize contamination of the root canal system during the procedure.	**1**
25	How important is aseptic technique for vital pulp therapy, nonsurgical RCT, revitalization?	Sterilization	ESE S3 2023	Expert Evidence-Based Rec No4	Maintain a meticulous aseptic technique, use sterile burs and limit waterline contamination, ensure optimal surgical field, apply dental dam isolation, use good light and magnification devices.	**1**

26	How important is aseptic technique for surgical endodontic treatment?	Sterilization	ESE S3 2023	Expert Evidence-Based Rec No5	Maintain a meticulous aseptic technique and optimal surgical field during surgery, use good light and magnification devices for improved visualization.	**1**
